# CK2β Is a Gatekeeper of Focal Adhesions Regulating Cell Spreading

**DOI:** 10.3389/fmolb.2022.900947

**Published:** 2022-06-29

**Authors:** Odile Filhol, Anne-Marie Hesse, Anne-Pascale Bouin, Corinne Albigès-Rizo, Florian Jeanneret, Christophe Battail, Delphine Pflieger, Claude Cochet

**Affiliations:** ^1^ Univ. Grenoble Alpes, INSERM, CEA, UMR Biosanté, U1292, Grenoble, France; ^2^ Univ. Grenoble Alpes, INSERM, CEA, UMR Biosanté U1292, CNRS FR 2048, Grenoble, France; ^3^ Univ. Grenoble Alpes, INSERM U1209, CNRS 5309, Institute for Advanced Biosciences (IAB), Grenoble, France

**Keywords:** CK2β depletion, EMT, tyrosine-phosphorylated proteins, FAK1-Src-PAX1 signaling pathway, focal adhesions, epithelial cell spreading

## Abstract

CK2 is a hetero-tetrameric serine/threonine protein kinase made up of two CK2α/αʹ catalytic subunits and two CK2β regulatory subunits. The free CK2α subunit and the tetrameric holoenzyme have distinct substrate specificity profiles, suggesting that the spatiotemporal organization of the individual CK2 subunits observed in living cells is crucial in the control of the many cellular processes that are governed by this pleiotropic kinase. Indeed, previous studies reported that the unbalanced expression of CK2 subunits is sufficient to drive epithelial to mesenchymal transition (EMT), a process involved in cancer invasion and metastasis. Moreover, sub-stoichiometric expression of CK2β compared to CK2α in a subset of breast cancer tumors was correlated with the induction of EMT markers and increased epithelial cell plasticity in breast carcinoma progression. Phenotypic changes of epithelial cells are often associated with the activation of phosphotyrosine signaling. Herein, using phosphotyrosine enrichment coupled with affinity capture and proteomic analysis, we show that decreased expression of CK2β in MCF10A mammary epithelial cells triggers the phosphorylation of a number of proteins on tyrosine residues and promotes the striking activation of the FAK1-Src-PAX1 signaling pathway. Moreover, morphometric analyses also reveal that CK2β loss increases the number and the spatial distribution of focal adhesion signaling complexes that coordinate the adhesive and migratory processes. Together, our findings allow positioning CK2β as a gatekeeper for cell spreading by restraining focal adhesion formation and invasion of mammary epithelial cells.

## Introduction

The serine/threonine protein kinase CK2 has been implicated in the phosphorylation of hundreds of cellular proteins, and more than 10% of the phosphoproteome matches the consensus sequence for CK2 phosphorylation (Meggio and Pinna, 2003; Salvi et al., 2009). CK2 exists as a tetrameric complex made up of two CK2α or CK2αʹ catalytic subunits and two CK2β regulatory subunits (Litchfield, 2003). Extensive studies have established that CK2 regulates the crosstalk among multiple signaling pathways critical for cell differentiation, proliferation, and survival (Ahmed et al., 2002; Litchfield, 2003; Hong and Benveniste, 2021; Roffey and Litchfield, 2021), thereby justifying its potential as a therapeutic target in diverse human diseases (Borgo et al., 2021; Strum et al., 2021). A variety of experimental evidence supports the view that deregulated CK2 is functionally linked to cancer (Chua et al., 2017; Nunez de Villavicencio-Diaz et al., 2017). For instance, CK2α, whose expression is abnormally high in a wide range of tumors, operates as a cancer driver by creating the cellular environment favorable to neoplasia (Guerra and Issinger, 2008; Kreutzer et al., 2010; Schmitt et al., 2021) and the CK2α gene (CSNK2A1) is one of 186 genes making up an “invasiveness signature” (Rozanov et al., 2008). CK2α overexpression in human breast cancers is predictive of metastatic risk and poor outcome (Giusiano et al., 2011). In contrast to other multi-subunit protein kinases, the free CK2α catalytic subunits possess a constitutive activity, while the homodimer of CK2β regulatory subunits operates as a regulatory component, modifying the accessibility of binding substrates to the catalytic site of the holoenzyme (Filhol et al., 2003; Filhol and Cochet, 2009; Deshiere et al., 2011). The spatiotemporal organization of the CK2 individual subunits in living cells coupled with the observation that the free CK2α subunit and the tetrameric holoenzyme have distinct, though overlapping, substrate specificity profiles has led to postulate that such a balance is crucial in the control of the many cellular processes that are governed by this pleiotropic kinase (Filhol et al., 2003; Filhol et al., 2004). Interestingly, a sub-stoichiometric expression of CK2β compared to CK2α in a subset of breast cancer tumors is correlated with the induction of hypoxia and epithelial to mesenchymal transition (EMT) markers, providing evidence that unbalanced expression of CK2 subunits may influence key cellular processes associated with epithelial cell plasticity in breast carcinoma progression (Deshiere et al., 2013; Golden and Cantley, 2014). We further demonstrate that CK2β depletion in non-transformed mammary epithelial cells induced dissociation of cell–cell contacts, led to the acquisition of a mesenchymal cell shape (properties described for EMT-induced cells), and drove breast cell stemness (Deshiere et al., 2008; Deshiere et al., 2011; Deshiere et al., 2013; Golden and Cantley, 2014; Filhol et al., 2015; Duchemin-Pelletier et al., 2017). These disparate effects may be dependent on the levels of free CK2α, which is markedly elevated in metastatic tumors compared to non-transformed cells (Kim et al., 2007; Laramas et al., 2007; Giusiano et al., 2011; Lin et al., 2011; Roelants et al., 2015; Choy et al., 2017; Schmitt et al., 2021). Phenotypic changes in cancer cells are controlled by a network of positive and negative regulators (Qian et al., 2015). In particular, phosphorylation-mediated signaling networks drive tumor progression and regulate inherent and acquired therapeutic resistance (Burridge, 2017). While tyrosine phosphorylation (pTyr) accounts for only 0.1–1% of the total phosphoproteome, 30% of the known oncoproteins are protein tyrosine kinases (PTKs) that regulate a vast array of cellular signaling networks necessary for processes such as survival, growth, migration, and invasion (Hunter, 1995; Blume-Jensen and Hunter, 2001; Olsen et al., 2006; Hochgrafe et al., 2010). Phenotypic changes in epithelial cells such as focal adhesion formation, cell spreading, and cytoskeletal reorganization are associated with pTyr signaling pathways. PTK activity can promote invadopodia formation, invasion, and diverse cellular processes implicated in EMT induction and subsequent steps in the metastatic cascade (Kim et al., 2014; Okayama et al., 2015). Since a CK2β level establishes a critical cell fate threshold in the control of epithelial cell plasticity, the purpose of this study was to evaluate the impact of an unbalanced expression of CK2 subunits on the potential activation of pTyr signaling pathways. We demonstrate that CK2β depletion in MCF10A mammary cells (ΔCK2β) promotes the phosphorylation on tyrosine residues of a number of proteins involved in cell migration and focal adhesion formation, including Src and focal adhesion kinase 1 (FAK1) tyrosine kinases. Our findings reveal that the unbalanced expression of the CK2 subunits, which was observed in a subset of breast tumors (Deshiere et al., 2013), may affect pTyr signaling cascades through activation of the FAK-Src-paxillin axis promoting spreading and migration of mammary epithelial cells.

## Material and Methods

### Cell Culture and Retroviral Infection

MCF10A cells from ATCC-LGS (Molsheim, France) (CRL-10317) are mammary epithelial cells derived from fibrocystic breast tissue from women with no family history of breast cancer and no evidence of disease. MCF7 cells are invasive breast cancer cells. Stable CK2β silencing was accomplished in MCF10A or MCF7 cells by transduction with lentiviruses pLKO1 (Sigma-Aldrich, St. Louis, MO, United States) followed by puromycin selection as previously described (Deshiere et al., 2013). Stable transfected cells were maintained in puromycin-containing medium for 5–6 days before use and the silencing efficiency was evaluated at the protein level by Western blot analysis. Mock-cells are MCF10A or MCF7 cells transduced with an empty pLKO1 vector and are referred in the article as WT MCF10A cells. They were both cultured as described (Debnath et al., 2003). Transient CK2β-siRNA transfection was performed as previously described (Deshiere et al., 2013).

### Western Blot Analysis

Western blot membranes were immunostained with primary antibodies as follows: phosphotyrosine 4G10 clone, #05-321 (Merck); P-FAK1 Y576/577, #3281, P-PAX1 Y118, #2541, P-p130CAS Y410, #4011, and P-EGFR Y1173, #4407 (Cell Signaling, Danvers, MA, United States); P-Src Y418, #11091 (SAB Signaling Antibody, Maryland, United States); Src, #sc-19, and EGFR, #sc-03 (Santa Cruz Biotechnology Inc., Santa Cruz, CA, United States); PAX1, #610052, FAK1, #610088, and p130CAS, #610271 (BD Biosciences, Pont de Claix, France); P-PLCγ Y771, #ab131455, and PLCγ, #ab52200 (Abcam, Paris, France); and GAPDH, #AM4300 (Invitrogen, Thermo Fisher, France). Secondary antibodies were peroxidase-conjugated affinity pure goat anti-rabbit IgG (#111035003) and peroxidase-conjugated affinity pure goat anti-mouse IgG (#115035003) from Jackson Immuno Research. Cells were scrapped in lysis buffer (50 mM Tris-HCl pH 7.4, 150 mM NaCl, 1% Triton X-100, 0.5% NP40, 1 M EDTA, 1 mM EGTA, 10 mM NaF, 2.5 mM Na_4_P_2_O_7_, 1 mM β-glycerophosphate, 1 mM Na_2_VO_4_, and protease-inhibitor cocktail, #P8340 (Sigma-Aldrich). The lysate concentrations were normalized after determination of protein concentration using the BCA protein assay kit (Thermo Scientific, Sacley, France). SDS-PAGE was performed using pre-cast 4–12% gradient gel (Bio-Rad, Hercules, CA, United States). Separated proteins at 20–25 μg/lane were transferred to PVDF membranes (100 V for 60 min). Blotted membranes were blocked during 1 h at room temperature with saturation buffer (1% BSA in 10 mM Tris-buffered saline, 0.1% Tween (TBST)) and then incubated with primary antibody diluted in saturation buffer, for 2 h or overnight. After three washes with TBST, appropriate secondary antibodies were added for 1 h at room temperature. Luminata Forte Western HRP substrate (Millipore, Billerica, MA, United States) was added and membranes were read with Fusion Fx7 (PerkinElmer, Waltham, MA, United States). Quantification was performed using ImageJ software.

### Immunoaffinity Purification of Tyr-Phosphorylated Cellular Proteins

Subconfluent WT and ΔCK2β MCF10A cells in two 10-cm culture dishes were washed with PBS and then lysed directly in lysis buffer (50 mM Tris-HCl pH 7.4, 150 mM NaCl, 1% Triton X-100, 0.5% NP40, 1 mM EDTA, 1 mM EGTA, 10 mM NaF, 2.5 mM Na_4_P_2_O_7_, 1 mM β-glycerophosphate, 1 mM Na_2_VO_4_, and protease-inhibitor cocktail) for 20 min on ice. After centrifugation at 14,000 *g* for 15 min, cell lysates were normalized for protein concentration and incubated overnight with 50 μl of anti-phosphotyrosine monoclonal antibody covalently coupled to agarose beads (4G10 resin). The beads were then washed three times with lysis buffer and twice with PBS. The adsorbed proteins were eluted in four successive washes of 100 μl containing100 mM phenyl phosphate in 10 mM Tris-HCl pH 7.5, 50 mM NaCl, and 2% glycerol.

### Phosphate Metal Affinity Chromatography of Phosphorylated Cellular Proteins

Phosphoprotein enrichment of cell extracts was performed according to the manufacturer’s protocol (TALON PMAC Phosphoprotein Enrichment Kit, Clontech Laboratories, United States). Subconfluent WT and ΔCK2β MCF10A cells in five 10-cm culture dishes were washed with PBS and then lysed directly in extraction/loading buffer A containing 10 mM NaF, 2.5 mM Na_4_P_2_O_7_, 1 mM β-glycerophosphate, 1 mM Na_2_VO_4_, and protease-inhibitor cocktail for 20 min on ice. After centrifugation at 14,000 *g* for 15 min, Na_2_VO_4_ which interferes with phosphoprotein binding to PMAC resin was removed by running the cell lysates through PD10 desalting columns. Samples of desalted cell lysates containing 3.5 mg protein were loaded on 1 ml of phosphoprotein affinity column (PMAC resin) and the column was agitated with the sample at 4°C for 2 h on a platform shaker. The column was then washed with 20 ml buffer A. The adsorbed proteins were eluted in three successive washes of 1 ml of buffer B (20 mM sodium phosphate and 0.5 M sodium chloride).

### Immunofluorescence

A total of 10,000 cells were seeded on glass coverslips coated with 2.5 μg vitronectin and incubated at 37°C 5% CO_2_ for 24 h. The cells were fixed with 4% PFA for 15 min at room temperature, washed three times with PBS, and permeabilized for 10 min with PBS containing 0.3% Triton X-100. After blocking with 3% BSA and 1% goat serum in PBS for 1 h, the cells were incubated with the primary antibody diluted in blocking solution for 1 h. The samples were then washed, incubated with a secondary Alexa Fluor-conjugated antibody (Thermo Fisher Scientific) for 1 h in blocking solution and Alexa-Fluor 647-phalloidin (A22287), when required, and finally washed and mounted using Mowiol-containing DAPI (Santa Cruz). Primary antibodies used for immunostaining are the following: P-tyrosine 4G10 (Millipore #05321), P-paxillin (Invitrogen #44-722G), P-FAK (Invitrogen #44-624G), and P-Src (Invitrogen #44-660G).

Images were acquired on an iMIC Andromeda (FEI, Gräfelfing, Germany) microscope at 40× magnification in spinning disk mode. We analyzed at least 50 cells per experiment in 3 independent experiments. Segmentation of focal adhesions was performed using Ilastik (interactive machine learning for (bio)image analysis) (Berg et al., 2019) and subsequently analyzed with Fiji software (Schindelin et al., 2012). Fiji is an open-source platform for biological image analysis. Statistics analyses were performed with R. Significant differences were evaluated by Student test.

### Protein Kinase CK2 Assay

Radiometric CK2 assay was performed in a final volume of 18 μl containing 3 μl of cell extract, and a mixture containing 100 μM of peptide substrate (RRREDEESDDEE), 10 mM MgCl_2_, and 100 μM [γ-^32^P]-ATP (6,000 Ci/mmol). Assays were performed at room temperature for 5 min before termination by the addition of 60 μl of 4% trichloroacetic acid. Incorporation of ^32^P in the peptide substrate was determined as previously described (Filhol et al., 1991). All kinase assays were performed in triplicates.

### Multiplex Kinase Activity Profiling

PamGene PTK multiplex activity assays were used to investigate TK activity of 196 PTKs. This platform utilizes a high-throughput peptide microarray system by measuring the phosphorylation of peptide representations of targets/substrates (referred hereafter as phosphosites) that are immobilized on the PamChip^®^ microarrays. Generic fluorescent-labeled antibodies that recognize phosphorylated residues are used to visualize the phosphorylation. Whole-cell lysates of WT and ΔCK2β MCF10A cells were prepared according to the manufacturer’s instructions (PamGene International, ’s-Hertogenbosch, the Netherlands). Cell samples were lysed for 30 min on ice and centrifuged for 15 min at 16,000 *g* at 4°C in a precooled centrifuge. After protein quantification (BCA protein determination, Pierce Scientific), aliquots of the samples were stored immediately in a −80°C freezer. Per array, 4 μg of protein and 100 μM ATP were loaded onto the appropriate PTK PamChip in kinase buffer. Phosphorylation of peptides was monitored by PamStation 12 following the manufacturer’s protocol as described previously (Arni et al., 2017). Technical triplicates were analyzed, and sequence-specific peptide tyrosine phosphorylation was detected by the fluorescein-labeled antibody PY20 (Exalpha, Maynard, MA). Capture of peptide phosphorylation signal is via a computer-controlled CCD camera. Kinomic profiling was performed using the Evolve software (PamGene International). Data were analyzed using the BioNavigator software version 6.3 (PamGene International) for raw data transformation into kinetic and steady-state values. Data were expressed as the average signal intensity (±SD) of the 196 peptide spots based on end levels of the phosphorylation curve. Prior to statistical analysis, fluorescent signal intensities were log2-transformed to satisfy the normality assumptions. Significant differences between two conditions were determined using two-sided Student’s *t*-test. The differential activity of kinases was predicted using an in-house bioinformatic approach, upstream kinase analysis (UKA) (Chirumamilla et al., 2019). Briefly, UKA identifies kinase activity based on a permutation analysis of the peptide substrate phosphorylation, by using known associations of phosphoproteins and kinases from literature and *in silico* databases. This permutation analysis gave a specificity score (mapping of peptides to kinases) and a significance score (kinase statistic that indicates the difference between treatment groups). Prior to UKA, the raw signal intensities were transformed using variance stabilizing normalization (VSN) (Huber et al., 2002).

### Mass Spectrometry-Based Proteomic Analyses

After phosphoprotein enrichment (both 4G10 and PMAC), the eluates were solubilized in Laemmli buffer before stacking of proteins in the top layer of a 4–12% NuPAGE gel (Invitrogen) for separation followed by R-250 Coomassie blue staining. The gel bands were manually excised and digested using modified trypsin (sequencing grade, Promega) as previously described (Salvetti et al., 2016). After peptide extraction, the samples were split into two parts before drying: one-third for proteome analysis and two-thirds for phosphopeptide enrichment. The phosphopeptides were enriched with TiO_2_ beads (GL Science) in batch mode with a modified protocol from Jensen and Larsen (2007). Briefly, the samples were mixed for 1 h with 0.6 mg beads in loading buffer (1 M glycolic acid in 80% acetonitrile (v/v) and 5% TFA (v/v)). The beads were washed three times with loading buffer, 80% acetonitrile with 1% TFA (v/v), and finally 10% acetonitrile (v/v) with 0.1% TFA (v/v). The phosphopeptides were eluted with 10% ammonia solution (v/v) for 10 min. After acidification with formic acid, the peptides were desalted using C18 ultra-micro spin columns (Harvard Apparatus) and dried under vacuum. The dried extracted peptides were resuspended in 5% acetonitrile and 0.1% trifluoroacetic acid and analyzed by online nano-liquid chromatography coupled to tandem mass spectrometry (LC–MS/MS) (Ultimate 3000 RSLCnano and the Q-Exactive HF, Thermo Fisher Scientific). The peptides were sampled on a 300-μm internal diameter, 5-mm length PepMap C18 precolumn (Thermo Fisher Scientific) and separated on a 75-μm internal diameter, 250-mm length C18 column (Reprosil-Pur 120 C18-AQ, 1.9 μm particles, Dr. Maisch HPLC GmbH). The column flow rate was 300 nL/min. The mobile phases consisted of solution A (water with 0.1% (v/v) formic acid) and solution B (acetonitrile with 0.1% (v/v) formic acid). For 4G10 samples, the peptides were eluted with a gradient consisting of an increase in solvent B from 5 to 13% in 1.5 min, then from 13 to 31% over 29 min, and from 31 to 41% over 4.5 min. For data-dependent acquisition (DDA), the spray voltage was set at 2 kV and the heated capillary was adjusted to 270°C. Survey full-scan MS spectra (*m*/*z* = 400–2,000) were acquired with a resolution of 60,000 after the accumulation of 3 × 10^6^ ions (maximum filling time 250 ms). The 12 most intense ions were fragmented by higher-energy collisional dissociation (HCD) after the accumulation of 1 × 10^6^ ions (maximum filling time 250 ms). For PMAC samples, the nano-LC method consisted of a 120-min multilinear gradient at a flow rate of 300 nl/min (same gradient slopes as for 4G10 samples). Survey full-scan MS spectra (*m*/*z* = 400–2,000) were acquired with a resolution of 60,000 after the accumulation of 1 × 10^6^ ions (maximum filling time 200 ms). The 20 most intense ions were fragmented by HCD after the accumulation of 1 × 10^5^ ions (maximum filling time 50 ms). MS and MS/MS data were acquired using the software Xcalibur (Thermo Scientific).

### Mass Spectrometry-Based Proteomic Data Processing

The data were processed automatically using Mascot Distiller software (version 2.7.1.0, Matrix Science). The peptides and proteins were identified using Mascot (version 2.8) through concomitant searches against Uniprot (*Homo sapiens* taxonomy, September 2021 version) and a database of 250 classical contaminants (homemade) and their corresponding reversed databases. Trypsin/P was chosen as the enzyme and two missed cleavages were allowed. Precursor and fragment mass error tolerances were set, respectively, to 10 and 20 ppm. Peptide modifications allowed during the search were cysteine carbamidomethylation (fixed), acetyl (protein N-terminal, variable), methionine oxidation (variable), and serine, threonine, tyrosine phosphorylation (variable). Proline software (version 2.1) (Bouyssie et al., 2020) was used to merge DDA results from proteome analysis and phosphopeptide enrichment. After combination, the results were filtered: conservation of rank 1 peptide-spectrum matches (PSMs) with a minimal length of 6 amino acids and a minimal score of 25. PSM score filtering was then optimized to reach a false discovery rate (FDR) of PSM identification below 1% by employing the target decoy approach. A minimum of one specific peptide per identified protein group was required. Proline was then used to perform MS1-based label-free quantification of the peptides and protein groups from the different samples with cross-assignment activated. Protein abundances were computed as a sum of specific peptide abundances, without using phosphopeptides and their counterparts. The data from 4G10 and PMAC enrichment were processed separately to obtain two different datasets.

### Statistical Analysis of Mass Spectrometry-Based Proteomic Data

Statistical analysis on the proteins from 4G10 dataset was performed using ProStaR (Wieczorek et al., 2017) to determine differentially abundant proteins between WT and ΔCK2β MCF10A cells. Protein sets were filtered out if they were not identified and quantified in at least two biological replicates of one condition. Reverse protein sets and contaminants were also filtered out. These filters downsized the dataset to 249 proteins. After log2 transformation, the data were normalized with VSN method. POV missing values were imputed with slsa method and MEC ones with 1-percentile value of each sample. Statistical testing was conducted using limma test. Differentially expressed proteins were sorted out using a log2 (fold change) cut-off of 1 and a *p*-value inferior to 0.05. Proteins with more than three imputed values in the upregulated condition were invalidated. For each replicate, pTyr-phosphopeptides were declared upregulated in ΔCK2β cells (or, respectively, WT cells) if they were detected only in this condition or if their log2 (fold change) was higher than 1 (respectively, lower than –1). The peptides upregulated in at least two replicates were further considered. For the PMAC protein dataset, after log2 transformation, missing values were imputed with 1-percentile value. Log2 (fold change) was calculated and normalized.

### Pathway Enrichment

Biological pathway enrichment analyses were carried out by Gene Set Enrichment Analysis (GSEA, FDR <0.05) from the 249 proteins quantified by proteomic analysis and kept during statistical analysis (Supplementary Table S1). The enrichGO and the cnet (category net plot used for visualization) functions were executed by ClusterProfiler v4.2.2 (Wu et al., 2015) (Bioconductor 3.14, https://www.bioconductor.org/, accessed on XX February 2022, R v4.1.2 (2021-11-01)). The enrichment analyses were performed using the biological pathway annotations from the Gene Ontology (GO, GO. db_v3.14.0 (2021-09-01), Bioconductor v3.14 Rv4.1.2 (2021-11-01), and KEGG databases (release 101, 2022/01) (Kanehisa and Goto, 2000). Gene sets consisting of at least 10 genes and less than 500 genes were retained for analysis. Thousand permutations were performed to compute *p*-values corrected by the Benjamini–Hochberg method.

## Results

### Changes in Tyrosine Kinase Activity in Extracts of WT and ΔCK2β MCF10A Cells

**FIGURE 1:**
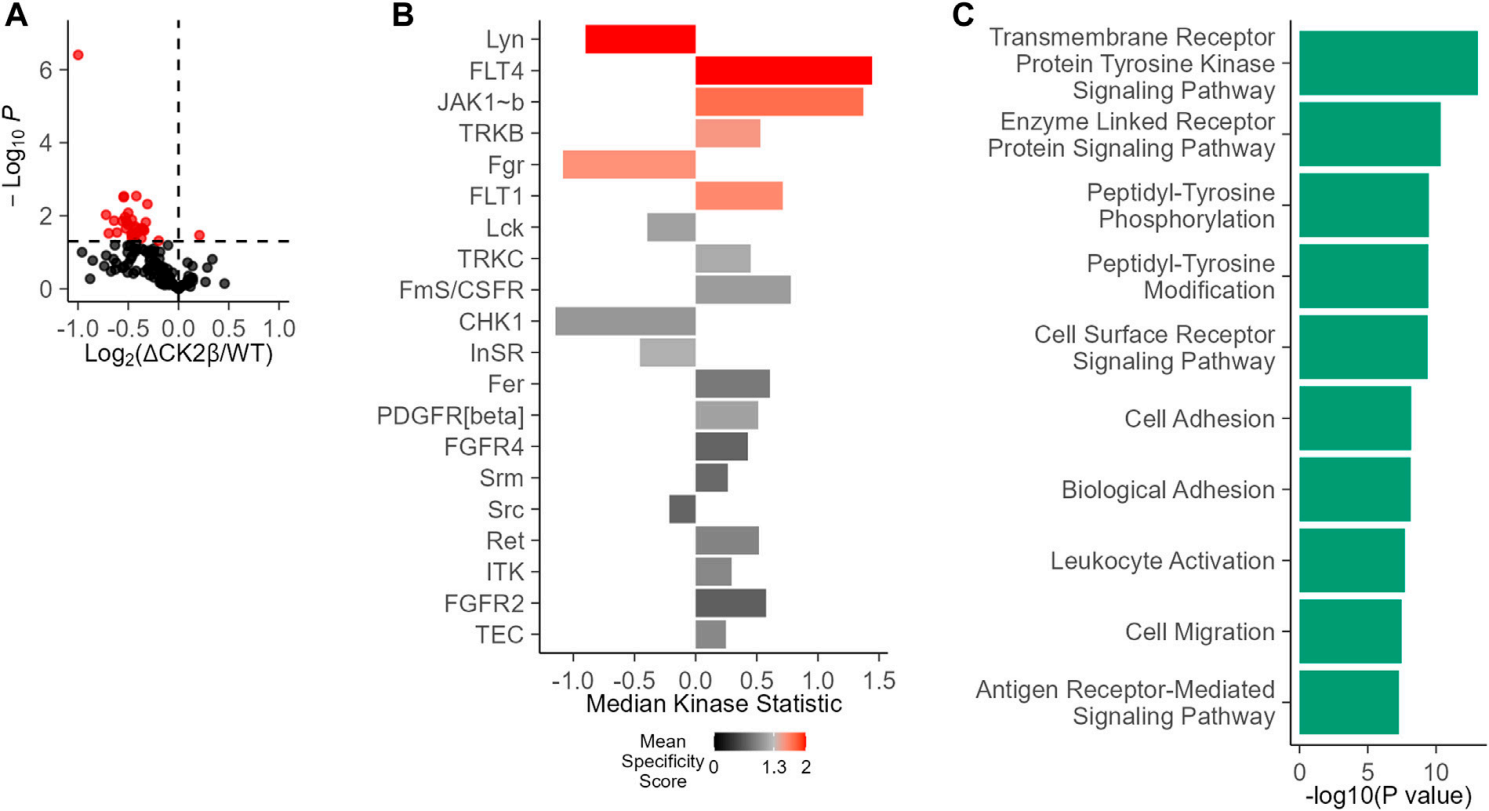
Changes in PTK activity in ΔCK2β MCF10A cells compared with WT cells. **(A)** Functional kinome profiling using the PamChips, which contain multiple copies of individual peptides. Sequence-specific tyrosine phosphorylation is detected by fluorochrome-coupled phospho-specific antibodies and CCD camera. The volcano plot visualizes the result of the tests by plotting, for each test, the effect size (*x*-axis, LFC or delta) versus significance (*y*-axis, –log10 (*p*-value)) of the test. Red spots are peptides that show significant differences compared to control (*p* < 0.05). **(B)** PTK score plot highlighting kinases that were modulated in ΔCK2β MCF10A cells compared with WT cells. The plot shows results from the upstream kinase analysis (UKA) tool, a functional scoring tool which rank-orders the top kinases that are differentially active between the two groups. The ranking factor is the final (median) kinase score. This score is based on a combined sensitivity score (difference between “treatment” and “control” groups) and specificity score (for a set of peptides to kinase relationship derived from current databases). The kinase statistics indicates the group differences, with effect size (values) and direction (+ or >0 = activation; – or 0 = inhibition). **(C)** Signaling pathways and biological process analysis in ΔCK2β MCF10A cells compared with WT cells (GProfiler, Gene Ontology Biological Processes).

Given the interplay between serine/threonine and tyrosine kinases, we evaluated the potential impact of an imbalanced expression of CK2 subunits on global tyrosine kinase activity in WT non-transformed mammary MCF10A or shRNA-mediated CK2β-silenced MCF10A cells (ΔCK2β) (Deshiere et al., 2013; Deshiere et al., 2011; Deshiere et al., 2008; Filhol et al., 2015; Duchemin-Pelletier et al., 2017; Golden and Cantley, 2014). For this purpose, we used the PamChip PTK multiplex activity profiling (PamGene International BV ’s-Hertogenbosch, Netherlands). This methodology has been developed as a screening tool that allows for the robust analysis of kinase activity from cells and tissues (Anderson et al., 2015; Arsenault et al., 2011). A 73% efficiency in CK2β silencing was evaluated at the protein level in ΔCK2β MCF10A cells (Supplementary Figure S1). Equal amounts of protein extracts of WT or ΔCK2β MCF10A cells were then applied to peptide array chips. In a two-group comparison between these two cell lines, the deregulation of phosphorylation for each peptide represents the changes in kinase activity. There was a clear difference in the mean pattern of peptide substrate phosphorylation on comparing WT and ΔCK2β MCF10A cells as illustrated in the volcano plot of Figure 1A, indicating that upon CK2β silencing, the activity of certain signaling pathways driven by distinct PTK was altered (Figure 1B). Based on those phosphorylation patterns, a computational upstream kinase analysis predicted increased activity of PTKs that are known to contribute to invasive progression and metastasis of breast tumor, namely, FLT4, JAK1b, TRKB, and FLT1 (Zhang et al., 2010; Qian et al., 2015; Choy et al., 2017; Wehde et al., 2018). In contrast, the activity of other TKs (Lyn and Fgr) was decreased in ΔCK2β MCF10A cells (Figure 1B). Thus, a significant change in the pattern of Tyr phosphorylation was noted when comparing WT and ΔCK2β MCF10A cells. A ranked list of possible canonical pathways (and networks) responsible for the differences in the peptide phosphorylation was established. CK2β loss induced changes in transmembrane PTK in correlation with biological events such as cell adhesion and migration (Figure 1C).

### Abundant pTyr-Containing Proteins Are Identified in ΔCK2β MCF10A Cells

**FIGURE 2:**
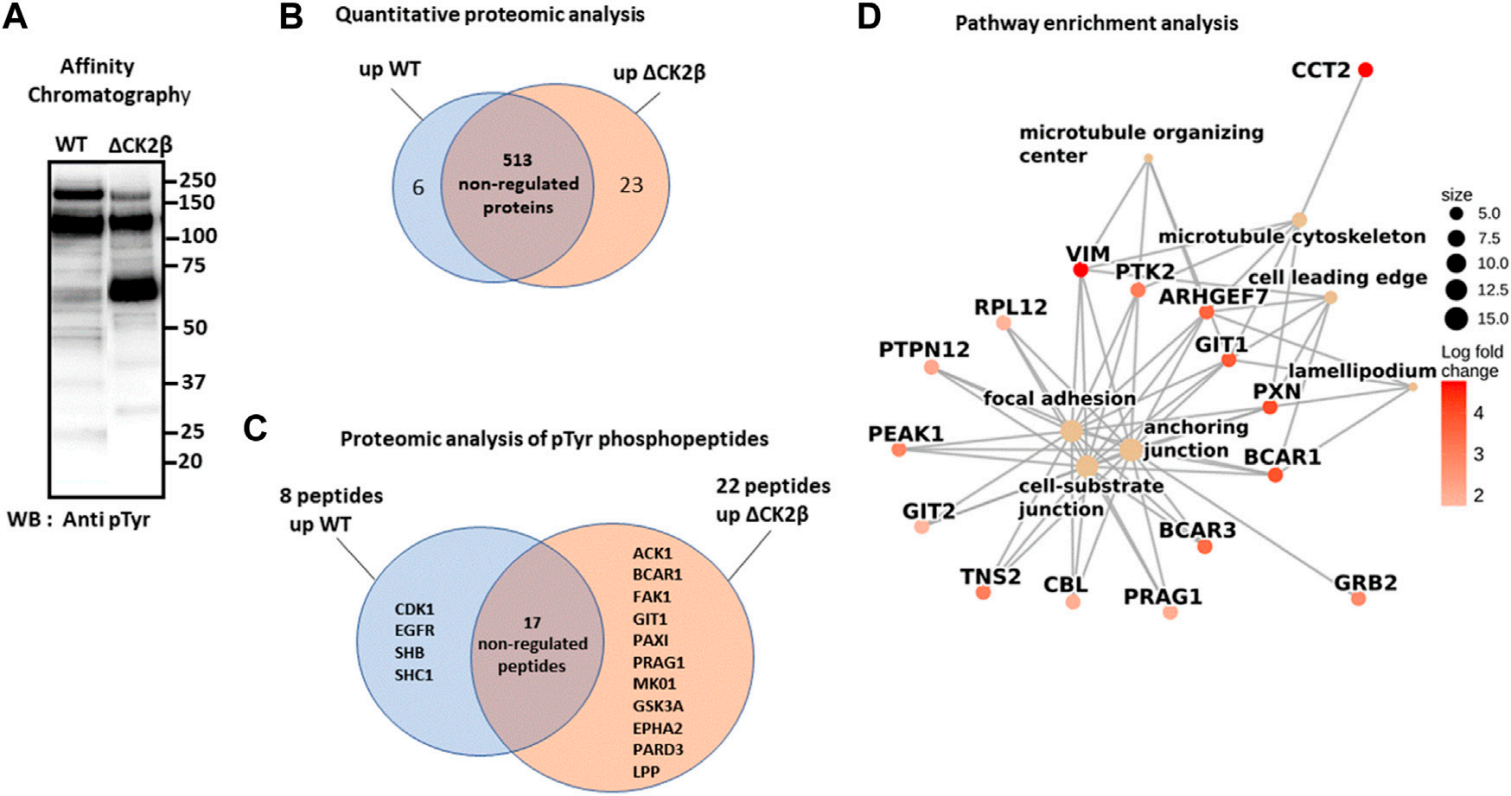
LC-MS/MS and GSEA analysis of differentially elevated levels of Tyr-phosphorylated proteins in WT and ΔCK2β MCF10A cells. **(A)** Proteins immunopurified from WT and ΔCK2β MCF10A cell lysates were analyzed by Western blotting for pTyr proteins using the 4G10 antibody. **(B)** Venn diagram showing differentially abundant proteins between WT and CK2β-depleted cells in samples obtained by immunoaffinity purification of Tyr-phosphorylated proteins (Supplementary Table S1). **(C)** Venn diagram showing the proteins in which differentially abundant pTyr-phosphopeptides between WT and CK2β-depleted cells were identified (Supplementary Table S2). These pTyr-phosphopeptides were identified and quantified from the LC-MS/MS analyses of peptide samples coming from a double enrichment starting from the two cell types: immunoaffinity purification of Tyr-phosphorylated proteins followed by the enrichment of their tryptic peptides on a TiO_2_ resin. **(D)** Significant gene ontology cellular components enriched from proteomics quantification of phosphoproteins (Supplementary Table S3) according to corrected *p*-values by the Benjamini–Hochberg method <0.05. Cellular component names are displayed as yellow nodes and bound to core phosphoproteins that led to a significant enrichment by GSEA. The core phosphoproteins are the nodes colored in red according to their log2 fold change value.

To obtain an exhaustive landscape of proteins differentially modified on Tyr residues in WT *versus* ΔCK2β MCF10A cells, we performed a proteomic analysis of pTyr-containing proteins. Since pTyr-containing proteins of low abundance might be difficult to detect within cell extracts, phosphoprotein enrichment was performed using pTyr immunoaffinity chromatography on anti-phosphotyrosine monoclonal antibody covalently coupled to agarose beads (4G10 resin). After extensive washing, the retained proteins were eluted with 100 mM phenyl phosphate, a phosphotyrosine hapten analog (Figure 2A). After trypsin digestion of the enriched proteins, a fraction of peptides was further loaded on a TiO_2_ resin, to allow the selection of phosphorylated peptides. We identified 542 unique proteins (contaminants filtered) from the total tryptic digest of the proteins retained on the 4G10 resin (Figure 2B, Supplementary Table S1). In addition, by analyzing the tryptic peptides further enriched on the TiO_2_ resin, 204 phosphosites were identified. Among them, 40 sites from 25 unique proteins were localized on tyrosine with a confidence higher than 75% (Supplementary Table S2). A large part of the pTyr-phosphopeptides was more abundant or specifically detected in ΔCK2β MCF10A cells (Figure 2C, Supplementary Table S2). This was the case not only for the EPH tyrosine receptor A2 (EPHA2) but also for a number of non-receptor tyrosine kinases including FAK1, the signaling pseudokinase pragmin (PRAG1), activated CDC42 kinase 1 (ACK1), glycogen synthase kinase 3α (GSK3A), and mitogen-activated protein kinase 1 (MK01). pTyr-phosphopeptides from signaling proteins, such as p130CAS/BCAR1, paxillin (PAX1), partitioning defective 3 (PARD3), lipoma-preferred partner (LPP), and ARF GTPase-activating protein (GIT1), that are activated by tyrosine phosphorylation, were also enriched or specifically detected in ΔCK2β MCF10A cells (Figure 2C). Interestingly, several of these well-characterized proteins form supra-molecular complexes belonging to adhesive structures at cell membrane known under the name of focal adhesions (FAs), playing important functional roles in integrin signaling (Wozniak et al., 2004; Burridge, 2017) and coordinating the adhesive and migratory processes (Geiger and Bershadsky, 2001). Among them, FAK1 as well as GIT1, ACK1, PAX1, p130CAS, PARD3, and LPP are known substrates of Src kinase (Wang et al., 2006; Sachdev et al., 2009; Meenderink et al., 2010; Totaro et al., 2014; Wu et al., 2015; Ngan et al., 2017; Atashrazm and Ellis, 2021) (Table 1). Of note, even if we did not identify upregulated pTyr-phosphopeptides from their sequences, several proteins such as vimentin (VIME), fibronectin 1 (FINC), sortin nexin-18 (SNX18), BCAR3 adaptor protein (BCAR3), tensin 2 tyrosine protein phosphatase (TNS2), inactive-tyrosine protein kinase PEAK (PEAK1), and growth factor receptor-bound protein 2 (GRB2) were also substantially enriched in ΔCK2β MCF10A cell extracts (Supplementary Table S1). In contrast, pTyr-phosphopeptides from the epidermal growth factor receptor (EGFR) and the SH2 domain-containing adapter protein B (SHB) were downregulated in ΔCK2β MCF10A cells suggesting a de-enrichment of the proliferation signature (Figure 2C, Supplementary Table S2).

**TABLE 1:** pTyr proteins enriched in ΔCK2β MCF10A and localized in adhesion complexes.

Name	pTyr Proteins	Adhesion Complex-Localized Proteins
FAK1	Focal adhesion kinase 1	Shen and Schaller (1999), Hu et al. (2014)
PRAG1	PEAK1-related kinase-activating pseudokinase	Senda et al. (2016), Abu-Thuraia et al. (2020)
ACK1	Activated CDC42 kinase	Modzelewska et al. (2006)
p130CAS	Crk-associated substrate	Harte et al. (1996), Donato et al. (2010), Machiyama et al. (2014)
PAX1	Paxillin1	Salgia et al. (1995), Brown et al. (1996)
GIT1	ARF GTPase-activating protein 1	Yin et al. (2005), Schmalzigaug et al. (2007)
Src	Proto-oncogene tyrosine protein kinase Src	Schaller et al. (1999), Volberg et al. (2001)
FLT1/4	Fms-related receptor tyrosine Kinase 1/4	Maru et al. (2001), Galvagni et al. (2010)
JAK1	Janus kinase 1	Petropoulos et al. (2016)
GSK3A	Glycogen synthase kinase 3 alpha	Kobayashi et al. (2006), Sun et al. (2013)
EPHA2	EPH receptor A2	Miao et al. (2000), Chen et al. (2018), Finney et al. (2021)
PARD3	Partitioning defective 3	Bridgewater et al. (2012), Valdivia et al. (2020)
LPP	Lipoma-preferred partner	Petit et al. (2003), Petit et al. (2005)

A pathway enrichment analysis by GSEA was carried out to identify deregulated molecular processes from proteomics quantification data (Supplementary Table S1). The enrichment analysis was performed from the log2 (fold change) values computed according to protein abundances between both phenotypes (ΔCK2β/WT). Figure 2D shows the seven significant cellular components enriched using the pathway annotation from the GO database (Supplementary Table S3). Significance was defined as each cellular component with a *p*-value less than 0.05 after the Benjamini–Hochberg correction. We observed that among them, the focal adhesion, anchoring junction, cell-substrate junction, microtubule cytoskeleton, and microtubule organizing center were significantly enriched in upregulated proteins. These enrichment insights support the idea that the overabundance of phosphoproteins involved in these biological processes following CK2β depletion leads to a revamping of the cell adhesion mechanisms. Moreover, the pathway enrichment by GSEA based on the KEGG database (Supplementary Table S4) reveals the upregulation of actin cytoskeleton as the only significant pathway. These results suggest that changes in phosphoprotein abundances following CK2β downregulation may also impair the actin or microtubule cytoskeleton regulation.

### Focal Adhesion Proteins Are Hyperphosphorylated in Extracts of ΔCK2β MCF10A Cells

**FIGURE 3:**
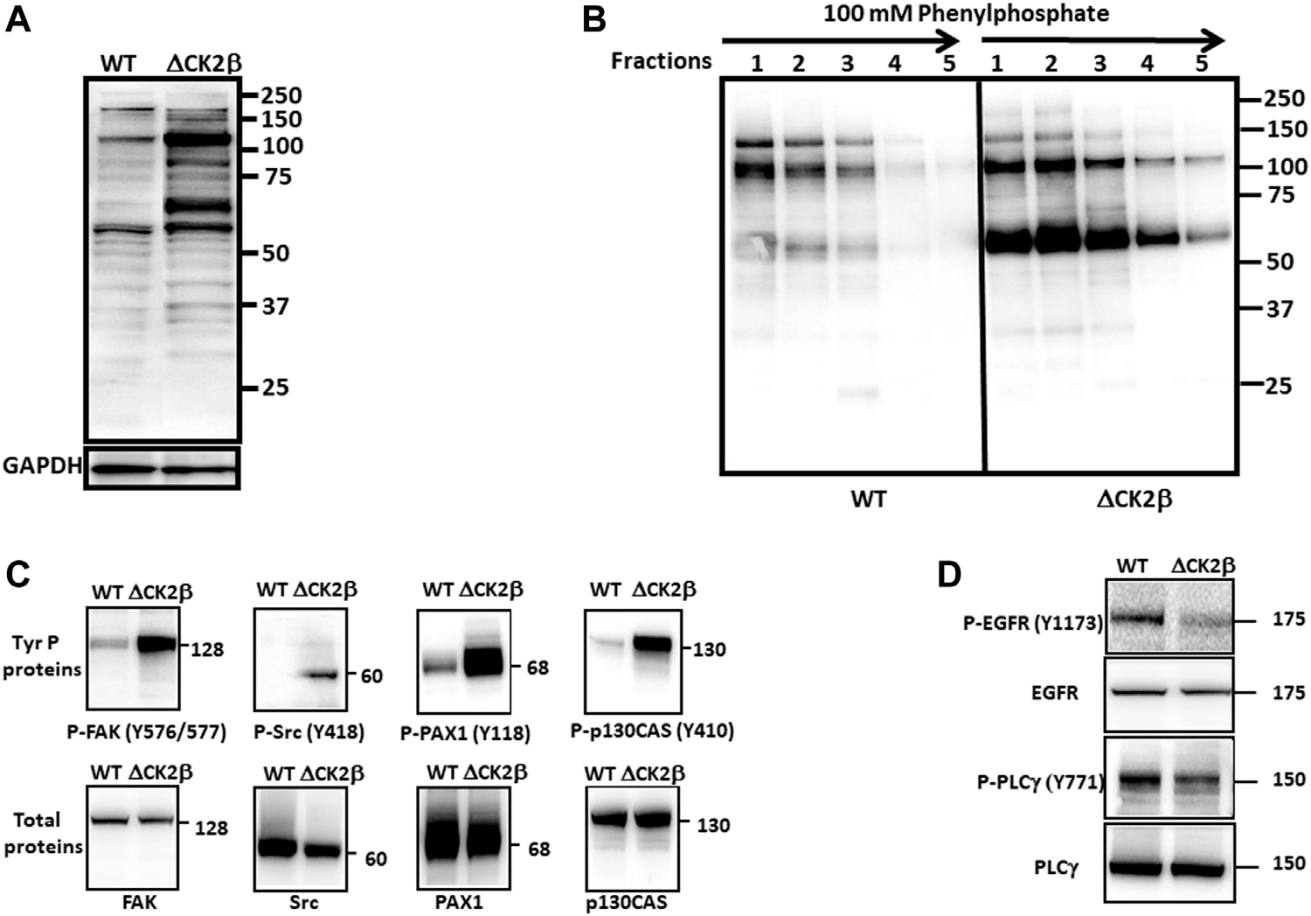
Upregulation of pTyr proteins in lysates of ΔCK2β MCF10A cells. **(A)** Lysate**s** from WT and ΔCK2β MCF10A cells were analyzed for pTyr-containing proteins using the 4G10 antibody. GAPDH was used as a loading control. **(B)** Cell extracts (500 μg of protein) of WT and ΔCK2β MCF10A cells were loaded on 4G10 resin. After extensive washing, the bound proteins were eluted with 100 mM phenyl phosphate. The collected fractions were analyzed by Western blotting with the 4G10 antibody. **(C)** Proteins immunopurified from WT and ΔCK2β MCF10A cell lysates were analyzed by Western blotting for p-FAK (Y576/577), p-Src (Y418), p-PAX1(Y118), p-p130CAS (Y410), FAK, Src, PAX1, and p130CAS and **(D)** for p-EGFR (Y1173), EGFR, p-PLCγ (Y771), and PLCγ.

In line with the phosphoproteomic approach, immunoblotting of cell extracts of subconfluent WT and ΔCK2β MCF10A cells with the 4G10 antibody confirmed the upregulation in CK2β-depleted MCF10A cells of diverse tyrosine-phosphorylated proteins (Figure 3A). Notably, Western blot analysis of pTyr proteins released from immobilized phosphotyrosine antibody (4G10 resin) highlighted the enrichment of two major upregulated phosphoproteins (65 and 115 kDa), most highly phosphorylated on Tyr residues in ΔCK2β cell extracts (Figure 3B). Similar patterns of enhanced tyrosine-phosphorylated proteins could be observed in either MCF10A cells which have been transiently depleted for CK2β or in shRNA-mediated CK2β-silenced MCF7 cells (Supplementary Figure 2 and Supplementary Figure S3). To go further, relevant antibodies also revealed the upregulation of additional pTyr-containing proteins in ΔCK2β MCF10A cells. Among them, non-receptor tyrosine kinases were identified, including FAK1 and the Src kinase. Important adhesion complex proteins such as PAX1 and p130CAS were also highly tyrosine phosphorylated (Figure 3C). Immunoblotting as well as immunoprecipitation analysis in these immunoaffinity-purified proteins suggested that the major 65-kDa tyrosine-phosphorylated protein may represent the scaffolding protein PAX1 (Supplementary Figure S4). PAX1 phosphorylation on tyrosine residue (Y118) is a prominent event during EMT and cell migration, through its recruitment into adhesion sites called FAs (Nakamura et al., 2000). PAX1 is also a well-known substrate for the FAK1/Src adhesion protein complex, which phosphorylates its residues Tyr31 and Tyr118 in dynamic adhesions, thus regulating both the assembly and the turnover of FAs (Schindelin et al., 2012). Plasma membranes act as transient trapping sites for signaling molecules playing a pivotal role in biological functions, including signal transduction and cell–ECM communication. Interestingly, it has been reported that the localization of a fraction of CK2 to the plasma membrane is controlled by cell–matrix interactions (Filhol et al., 2015). Moreover, protein–protein interaction between the scaffold protein CKIP-1 and CK2 provided evidence for CK2 targeting to the plasma membrane (Olsten et al., 2004). Therefore, selected differentially tyrosine-phosphorylated proteins known to be localized to the plasma membrane were validated using immunoblotting. This was the case for the activation of EGFR, a key driver in growth factor-dependent signal transduction, and its downstream effector PLCγ in WT and ΔCK2β MCF10A cells. The phosphorylation of EGFR and PLCγ on Tyr1173 and Tyr771, respectively, was decreased in ΔCK2β MCF10A cells compared to WT cells (Figure 3D). Thus, in agreement with the proteomic analysis (Figure 2D), CK2β downregulation significantly inhibited the activated form of EGFR and reduced the activation of its downstream signaling molecule PLCγ. Collectively, these data were consistent with the proteomic analyses, highlighting the phosphorylation-dependent activation of focal adhesion markers of the FAK1-Src-PAX1 axis upon CK2β depletion in MCF10A cells.

### CK2β Depletion Induces Cell Spreading and Increase of Focal Adhesion Number

**FIGURE 4:**
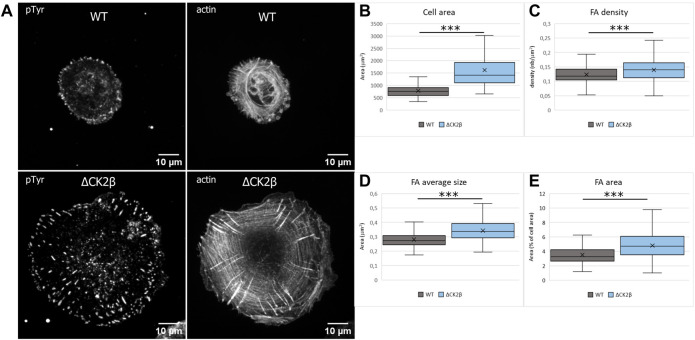
Cell spreading and size of focal adhesions are dependent on CK2β. **(A)** Staining of pTyr and actin was carried out on cells spread on vitronectin-coated cover glass for 24 h. Scale bar represents 10 µm. **(B)** Quantification of the cell area. **(C)** Quantification of the number of FAs normalized to the cell area. **(D)** Quantification of the FA average area. **(E)** Quantification of the FA area, normalized to the cell area. Error bars represent standard deviation. ***p-value ≤0.0005.

Early evidence indicated that tyrosine phosphorylation plays an important role in the overall organization of adhesion complexes and their dynamic regulation (Craig and Johnson, 1996; Burridge et al., 1988). In addition, tyrosine phosphorylation events were acknowledged to be concentrated at focal adhesions formed by the cells with the substratum (Maher et al., 1985). It has also been reported that PAX1 and FAK1 enter nascent adhesions as discrete entities and form dynamic molecular complexes within adhesion sites. Moreover, phosphorylation of PAX1 at Y31-Y118 regulates the formation and size of the complexes (Choi et al., 2011). As these focal adhesion markers were identified in ΔCK2β MCF10A cells after pTyr immunoaffinity chromatography, we analyzed the impact of CK2β depletion in the distribution of focal adhesions of isolated MCF10A cells spread onto vitronectin. Vitronectin was chosen as the adhesive substrate since WT or ΔCK2β MCF10A cells plated on vitronectin-coated coverslips clearly display a distinguishable morphology (Deshiere et al., 2013). In particular, ΔCK2β MCF10A cells plated on vitronectin-coated coverslips display higher cell spreading as evidenced by the projected area of actin cytoskeleton (Figure 4A and Figure 4B). This cell spreading is associated with an increase in the number (Figure 4C) and the size (Figure 4D) of focal adhesions giving rise to an increase in the total adhesive surface when compared to the cell surface (Figure 4E). FAK1 phosphorylation is one of the key steps following integrin engagement, either directly or through its role as a scaffolding protein for Src (Tapial Martinez et al., 2020). Based on the increase in cell spreading upon CK2β loss, we investigated whether this increase might be related to a change in focal adhesion organization by immunostaining FAK1, Src, and PAX1 (Supplementary Figure S5A,F,K). Immunofluorescence analyses demonstrated that CK2β depletion increased the Src labeling and changed the spatial distribution of FAK1 and PAX1 in focal adhesion sites. Morphometric analyses showed that CK2β-depleted cells were much larger than WT cells. Moreover, descriptors of focal adhesion morphology such as number and mean size of the focal adhesions were strikingly upregulated in these cells (Supplementary Figure S5). It has been reported that the mean size of focal adhesions uniquely predicts cell speed independently of focal adhesion surface density and molecular composition (Kim and Wirtz, 2013). The regulation of FAs by CK2β is consistent with our previous observations, showing that CK2β-depleted cells displayed a mesenchymal phenotype and a loss of polarity driving a 2D-to-3D morphologic transformation (Deshiere et al., 2008; Deshiere et al., 2013).

### The Catalytic Activity of CK2 Is Not Involved in Enhanced Tyr Phosphorylation in ΔCK2β MCF10A Cells

**FIGURE 5:**
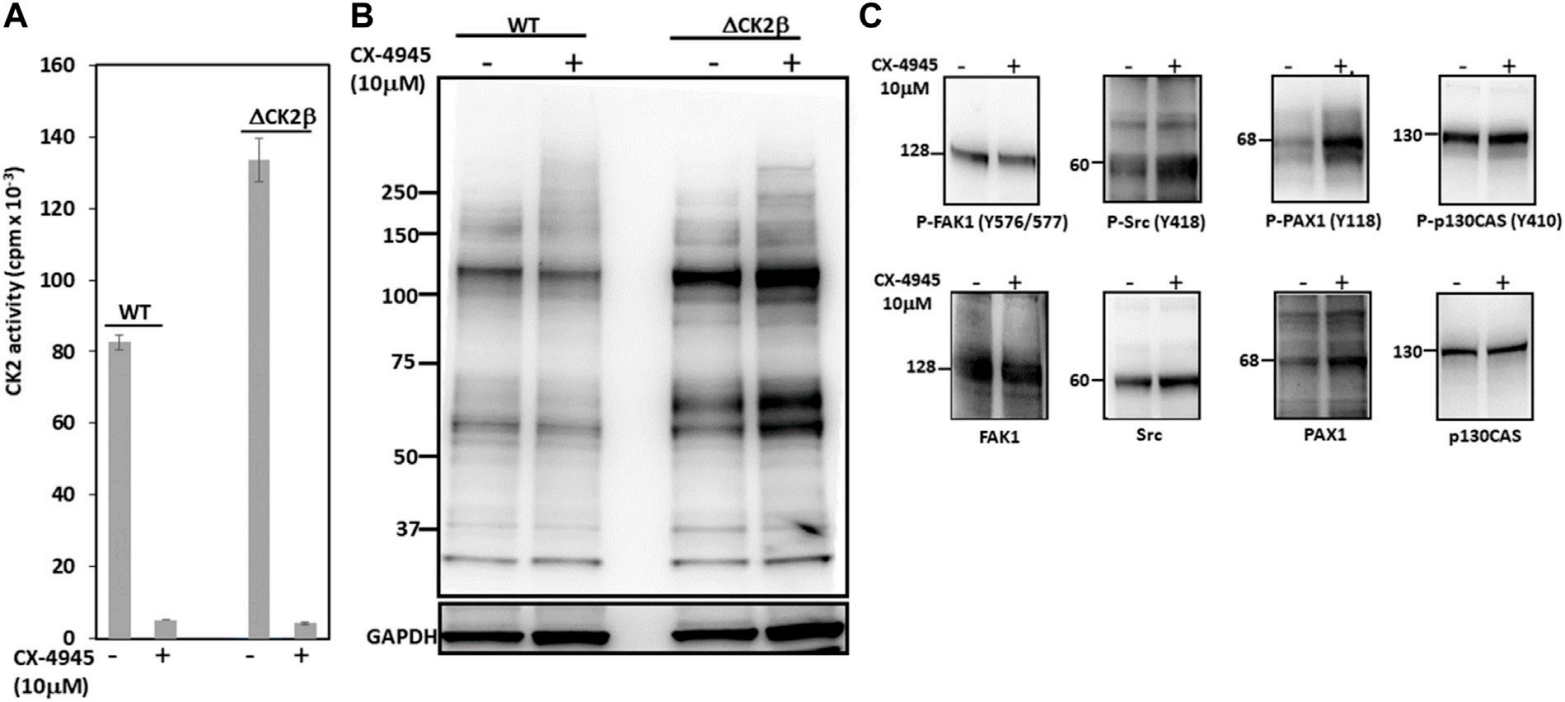
CK2 catalytic activity does not contribute to enhanced Tyr phosphorylation in ΔCK2β MCF10A cells. **(A)** CK2 kinase activity in cell lysates from WT or ΔCK2β MCF10A cells treated for 5 h in the absence or presence of 10 μM CX-4945. **(B)** Cell lysates as in **(A)** were Western blotted with 4G10 antibody for the presence of pTyr proteins. GAPDH was used as a loading control. **(C)** ΔCK2β MCF10A cell lysates were also analyzed by Western blotting for p-FAK (Y576/577), p-Src (Y418), p-PAX1 (Y118), p-p130CAS (Y410), FAK, Src, PAX1, and p130CAS.

CK2 is mostly known as a Ser/Thr kinase, yet the enzyme also exhibits tyrosine kinase activity in mammalian cells (Basnet et al., 2014; Borgo et al., 2021). To clarify the potential contribution of CK2 activity in the activation of these Tyr phosphorylations, we analyzed pTyr proteins in the extracts of WT or ΔCK2β MCF10A cells that had been incubated for 5 h with 10 μM of the small chemical CK2 inhibitor CX-4945. CK2 activity measured in whole-cell lysates from WT or ΔCK2β MCF10A cells treated with CX-4945 was inhibited by more than 90% (Figure 5A). Immunoblotting analysis of protein extracts from cells treated under these conditions revealed that the inhibition of CK2 did not abrogate the Tyr phosphorylation of the 65- and 115-kDa endogenous proteins (Figure 5B). In addition, the Tyr phosphorylation of adhesion complex proteins was either not affected (FAK1, p130CAS) or enhanced (Src, PAX1) upon CX-4945 treatment (Figure 5C). The increased Tyr phosphorylation of Src and PAX1 observed in ΔCK2β MCF10A cells after CX-4945 treatment suggests that the activation of these two components of the adhesion complexes could be similarly antagonized by CK2β and CK2α. This would reinforce the notion that any variation in the expression/activity of either CK2 subunits could have significant impacts on focal adhesion signaling pathways.

## Discussion

In the present study, we demonstrate that CK2β downregulation in MCF10A mammary cells triggers the phosphorylation of an array of tyrosine-phosphorylated proteins involved in cell migration and focal adhesion including Src and FAK1 tyrosine kinases. Our findings highlight a crosstalk between CK2, a serine/threonine kinase, and pTyr signaling pathways involved in phenotypic changes of epithelial cells. Such a potential link has been previously evoked. For instance, the catalytic subunits of CK2 are readily phosphorylated *in vitro* by the Src family protein tyrosine kinases Lyn and c-Fgr with a concomitant threefold increase in catalytic activity of CK2 (Donella-Deana et al., 2003). Interestingly, the presence of CK2β decreased this Lyn/Fgr-mediated phosphorylation, suggesting that at least *in vitro*, the CK2 holoenzyme is less prone to tyrosine phosphorylation. One could speculate that, within cells, the pool of catalytic subunits that is not incorporated into the holoenzyme would acquire an increased activity toward a subset of specific targets. Indeed, it has been reported that in advanced cancers, the TGFβ receptor (TGFBR) 1 kinase phosphorylated CK2β targeting it for degradation. The resulting CK2α/CK2β subunit imbalance promoted the activation of CK2 and EMT induction (Kim et al., 2018). Thus, a dysregulation in CK2 subunit levels, originating either from shRNA-mediated CK2β silencing or from TGFβ-dependent decrease of CK2β, invariably promotes EMT activation in epithelial cells (Deshiere et al., 2013; Filhol et al., 2015). Consequently, it is conceivable that signaling-mediated drop of CK2β expression could increase intracellular CK2 activity impacting downstream signaling events such as pTyr-relayed signaling cascades. Indeed, our results reveal that spreading-coupled pTyr signaling pathways including FAK1, PAX1, ACK1, EPHA2, PARD3, and LPP are impacted by CK2β depletion (Supplementary Table S2). Of note, the focal adhesion network (www.adhesome.org) which is involved in the regulation of cell shape and focal adhesion complexes is acting at the plasma membrane level. This is consistent with previous observations showing that in epithelial cells, the association of a fraction of CK2 to the plasma membrane is controlled by cell–matrix interactions (Deshiere et al., 2008). Phosphoproteomic analysis revealed that FAK1 is activated through membrane recruitment by growth factors, extracellular matrix, and integrin signaling followed by subsequent autophosphorylation at Tyr 397 exposing an SH2-binding domain, which in turn recruits Src and promotes phosphorylation of FAK1 at Tyr 576/577 (Robertson et al., 2015). The fully active FAK1/Src complex can then recruit, phosphorylate, and activate numerous targets including p130CAS and PAX1, which play critical roles in regulating cell spreading, cell motility, and cytoskeletal modifications (Zhang et al., 2010). In this line, it has been shown that maximal tyrosine phosphorylation of PAX1 by Src and FAK1 is required for the induction of anchorage-independent signal transduction, a characteristic of metastatic cells (Vilk et al., 2008; Anderson et al., 2015). As a signal transducer for ECM–tumor cell interactions, FAK1 expression and/or activation has been found altered in most human epithelial cancers, resulting in enhanced invasive potential and poor overall patient survival (McLean et al., 2005; Luo and Guan, 2010). Similarly, CK2α overexpression in a significant fraction of breast cancers is predictive of metastatic risk (Giusiano et al., 2011). Moreover, suppression of CK2β expression in a subset of breast cancers modulates the expression of EMT-related markers (Deshiere et al., 2013). Our findings now demonstrate that a loss of CK2β leads to changes in the size of the focal adhesions, through the activation of the well-characterized FAK1-Src-PAX1 signaling pathway.

While CK2β is traditionally considered as a regulatory subunit of CK2, some studies also suggest that this subunit might function independently of CK2α (Filhol et al., 2015; Borgo et al., 2019; Lettieri et al., 2019). Indeed, blocking the CK2 catalytic activity in CK2β-depleted cells with CX-4945 did not affect the Tyr phosphorylation of FAK1 and p130CAS while triggering enhanced phosphorylation of Src and PAX1, supporting the hypothesis that CK2β may play additional roles outside of the CK2 tetrameric holoenzyme (Bibby and Litchfield, 2005; Borgo et al., 2019). Our data show that by limiting the focal adhesion formation, CK2β appears as a major molecular break for cell spreading. The impact of CK2β on FAs is supported by the role of CK2 in cytoskeletal alteration (Canton and Litchfield, 2006; Lettieri et al., 2019) and in the regulation of actin and tubulin polymerization (reviewed in D'Amore et al., 2019). For instance, it has been previously reported that epithelial cell polarity and morphology might be controlled by CK2 through the phosphorylation of the Pleckstrin homology domain-containing protein (CKIP-1) and coronins, key proteins playing roles in the functional organization of actin-dependent cellular processes like protrusion formation, migration, and invasion (Canton et al., 2005; Deshiere et al., 2008; Deshiere et al., 2011; Xavier et al., 2012; Filhol et al., 2015).

We have generated proteomics data on samples enriched by PMAC (Supplementary Table S5), which give a global overview of the phosphoproteome, and on the 4G10 affinity resin which enriches for pTyr-modified proteins (Supplementary Table S2). Both approaches provided a complementary view on the phosphoproteins deregulated upon deletion of the CK2β subunit and are also incomplete, as they missed many proteins involved in the Src pathway, for example. To follow specifically the phosphorylation state of proteins of interest, a complementary approach such as targeted proteomics on the proteolytic peptides containing the modified Ser/Thr/Tyr residues of interest or Western blot analysis using specific antibodies would be better suited. Yet targeted proteomics requires time for method development and Western blot analysis is only possible if antibodies of validated specificity are available.

In summary, the data collected here support the notion that CK2β works by more than altering the CK2 activity: it also plays a key role in the negative regulation of focal adhesion maturation, blocking dominant pTyr downstream signaling pathways involved in cell scattering and invasion. Further functional characterization of the molecular mechanism by which CK2β could restrain these pathways in normal human mammary cells is the subject of ongoing work. More broadly, this analysis will cast additional insights into the specific contribution of individual CK2 subunits during breast carcinoma progression.

## Data Availability

Publicly available datasets were analyzed in this study. The LC-MS/MS data have been submitted to the ProteomeXchange Consortium via the PRIDE (Perez-Riverol et al., 2019) partner repository under dataset identifier PXD030991.
