# Supplementary material for: CK2β Is a Gatekeeper of Focal Adhesions Regulating Cell Spreading
Source: Front Mol Biosci. 2022 Jun 29;9:900947. doi: 10.3389/fmolb.2022.900947 (PMC9280835; doi:10.3389/fmolb.2022.900947)
Supplement: Supplementary file 1 [file DataSheet1.zip › Sup data/legends for suppl. figures.docx]

**Supplementary data**

***Sup. Figure 1. Stable CK2β knock-down in MCF10A cells***.  *Lysate****s*** *from* *WT and Δ*CK2β *MCF10A cells were analyzed* *for CK2α and* CK2β *expression by Western blotting. HSP90 was used as a loading control.*

***Sup. Figure 2. Transient*** ***CK2β knock-down in MCF10A cells***. ***(A)*** *Lysate****s*** *from* *MCF10A cells transiently transfected with Ctrl or* CK2β *siRNA were analyzed for CK2α and* CK2β *expression; (****B****) for phosphotyrosine-containing proteins; (****C****) for p-PAX1 (Y118) and PAX1 expression.*

*HSP90 or GAPDH were used as loading controls.*

***Sup. Figure 3. Stable CK2β knock-down in MCF7 cells***. ***(A)***  *Lysate****s*** *from* *WT and Δ*CK2β *MCF7 cells were analyzed for* CK2β *expression or* ***(B)*** *phosphotyrosine-containing proteins. HSP90 was used as loading control.*

***Sup. Figure 4. PAX1 is the main tyrosine phosphorylated protein in extracts of ΔCK2β MCF10A cells. (A)*** *Proteins eluted from* *4G10 resin were analyzed by Western blotting for pTyr-containing proteins using the 4G10 or p-PAX1 (Y118) antibodies.* ***(B)*** *Proteins as in* ***(A)*** *were immunoprecipitated using an anti-PAX1 antibody. After immunoprecipitation, proteins present in the supernatant (Sup) or in anti-PAX1 antibody beads were western blotted with 4G10 or p-PAX1 (Y118) antibodies.*

***Sup. Figure 5. CK2β depletion leads to changes in focal adhesion organization. A; F; K.*** *Staining of pFAK1 (****A****), pSrc (****F****) pPAX1 (****K****) and actin was carried out on indicated cells spread on vitronectin-coated coverglass for 24 h. Scale bar represents 10 µm.* ***B; G; L.*** *Quantification of the cell area.* ***C; H; M.*** *Quantification of the number of FA normalized to the cell area.* ***D; I; N.*** *Quantification of the FA average area* ***E; J O.*** *Quantification of the FA area, normalized to the cell area. Error bars represent standard deviation. *** p.value ≤ 0.0005.*

***Sup Table 1***

*MS-based proteomic characterization and quantification of proteins from WT and CK2β-depleted cells in samples obtained by immunoaffinity purification of Tyr-phosphorylated proteins. Differentially abundant proteins are represented in individual tabs.*

***Sup Table 2***

*MS-based proteomic characterization of phosphosites from WT and CK2β-depleted cells in samples obtained by immunoaffinity purification of Tyr-phosphorylated proteins. Quantification and differentially abundant pTyr-phosphopeptides are represented in individual tabs.*

***Sup Table 3***

*go_gse_CellularComponent.csv. Pathway enrichment results of Tyr-phosphorylated proteins values from GSEA analysis based upon the GO Celullar Component database****.***

***Sup Table 4***

*kegg_gse.csv. Pathway enrichment results of Tyr-phosphorylated proteins values from GSEA analysis based upon the KEGG database****.***

***Sup Table 5***

*MS-based proteomic characterization and quantification of proteins from WT and CK2β-depleted cells in samples obtained by global PMAC phosphoprotein enrichment.*
