# Supplementary figures and images for: CK2β Is a Gatekeeper of Focal Adhesions Regulating Cell Spreading
Source: Front Mol Biosci. 2022 Jun 29;9:900947. doi: 10.3389/fmolb.2022.900947 (PMC9280835; doi:10.3389/fmolb.2022.900947)

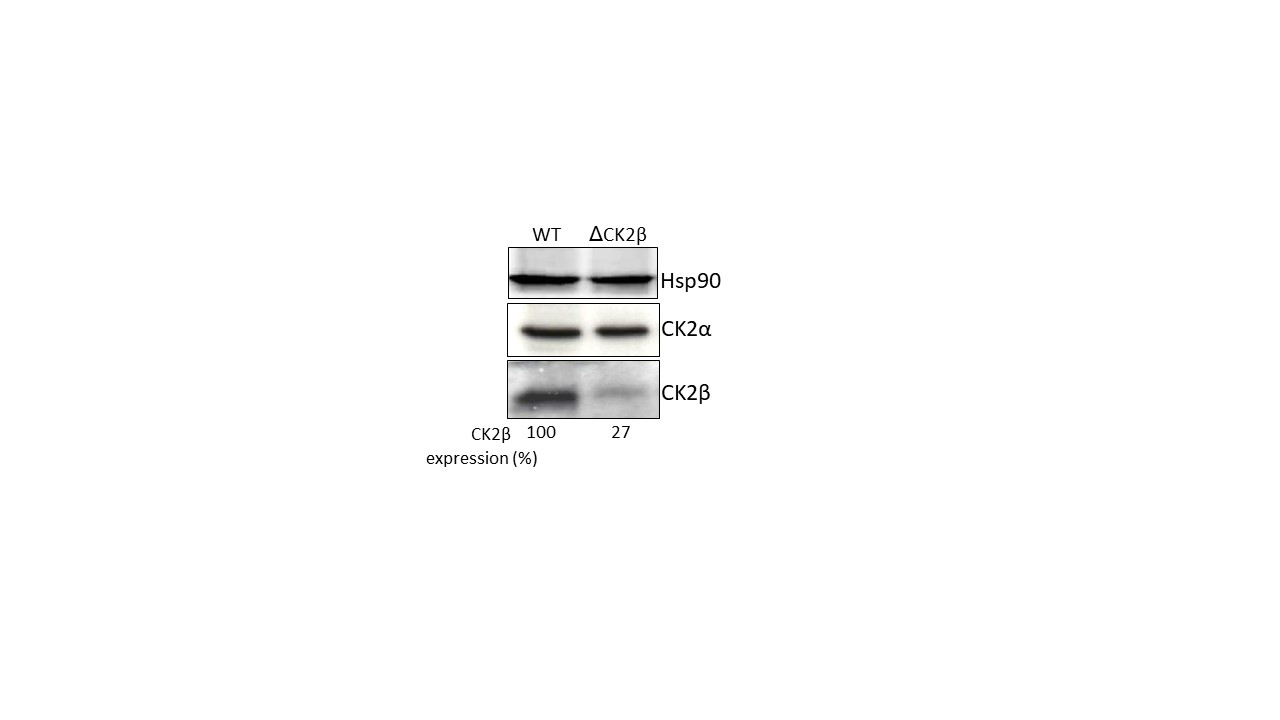

Supplement: Supplementary file 1 [file DataSheet1.zip › Sup data/Sup Fig 1.jpg]

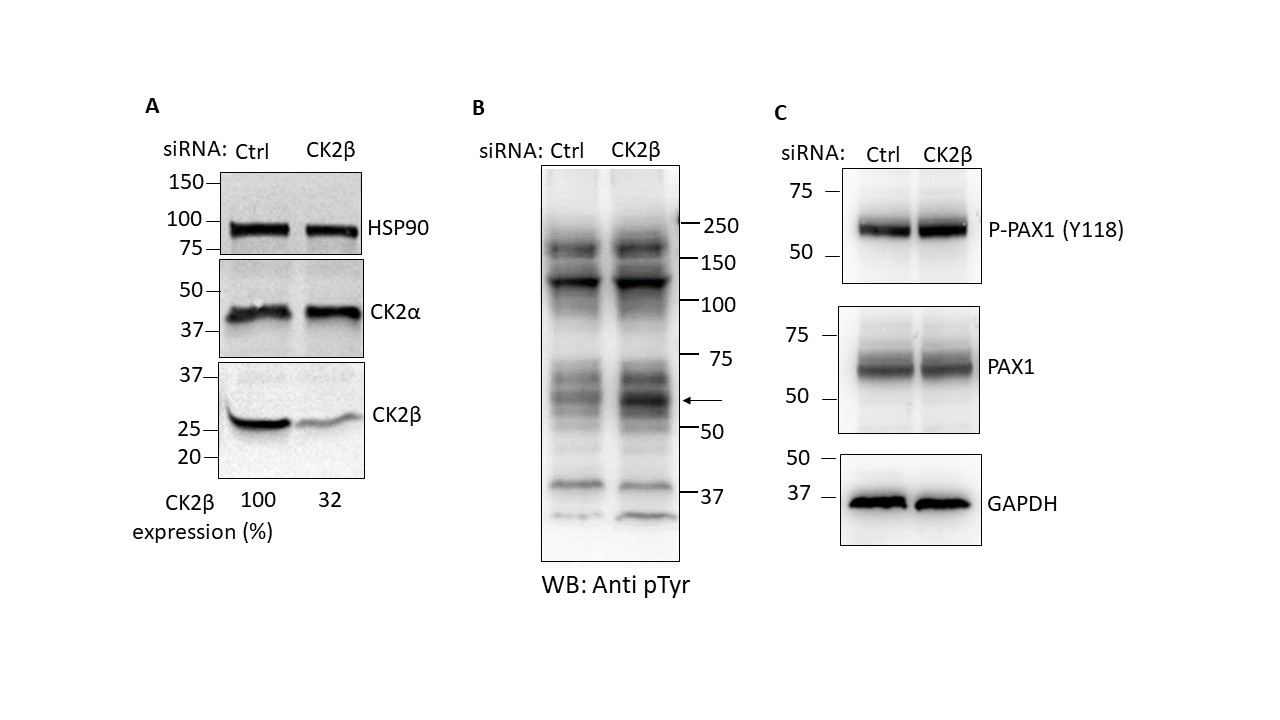

Supplement: Supplementary file 1 [file DataSheet1.zip › Sup data/Sup Fig 2.jpg]

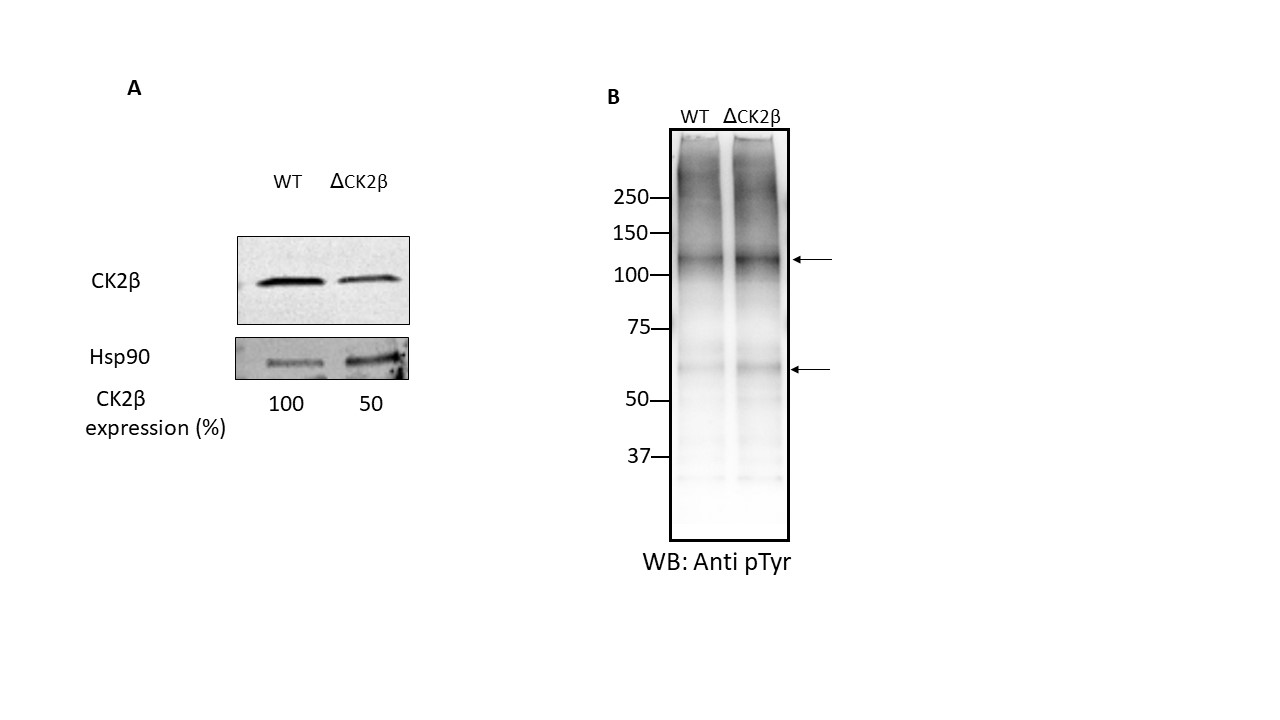

Supplement: Supplementary file 1 [file DataSheet1.zip › Sup data/Sup Fig 3.jpg]

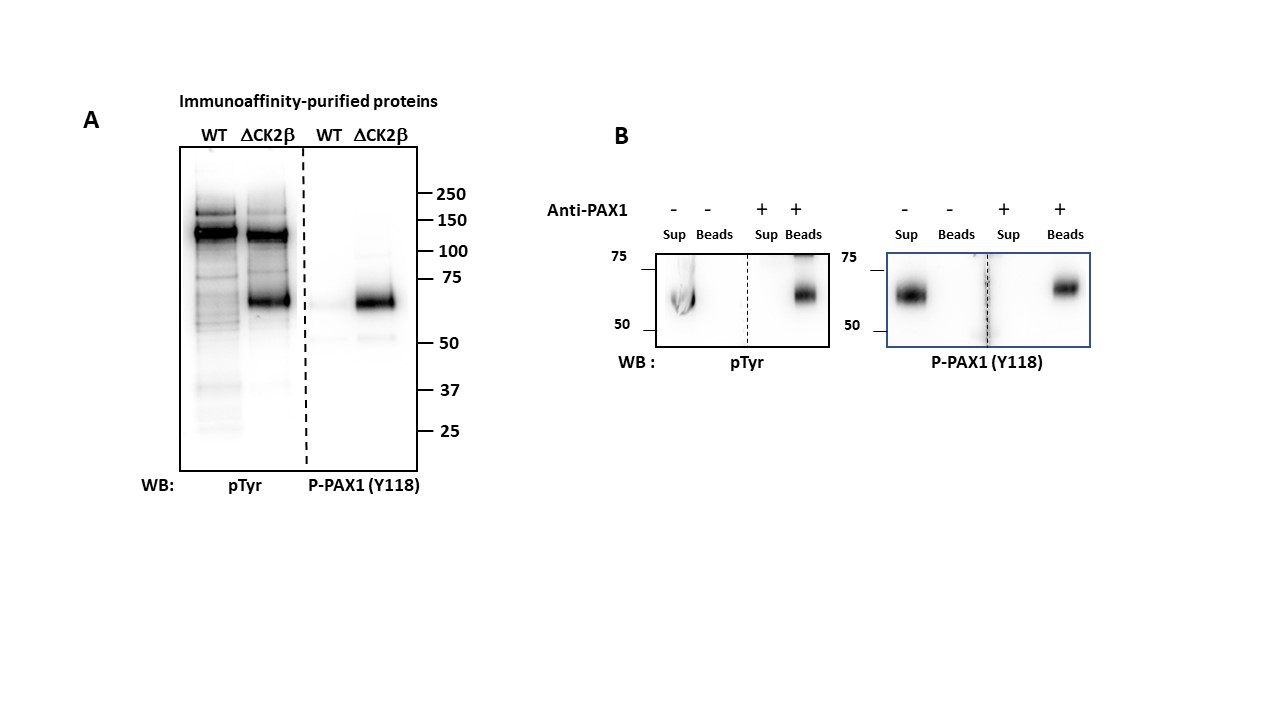

Supplement: Supplementary file 1 [file DataSheet1.zip › Sup data/Sup Fig 4.jpg]

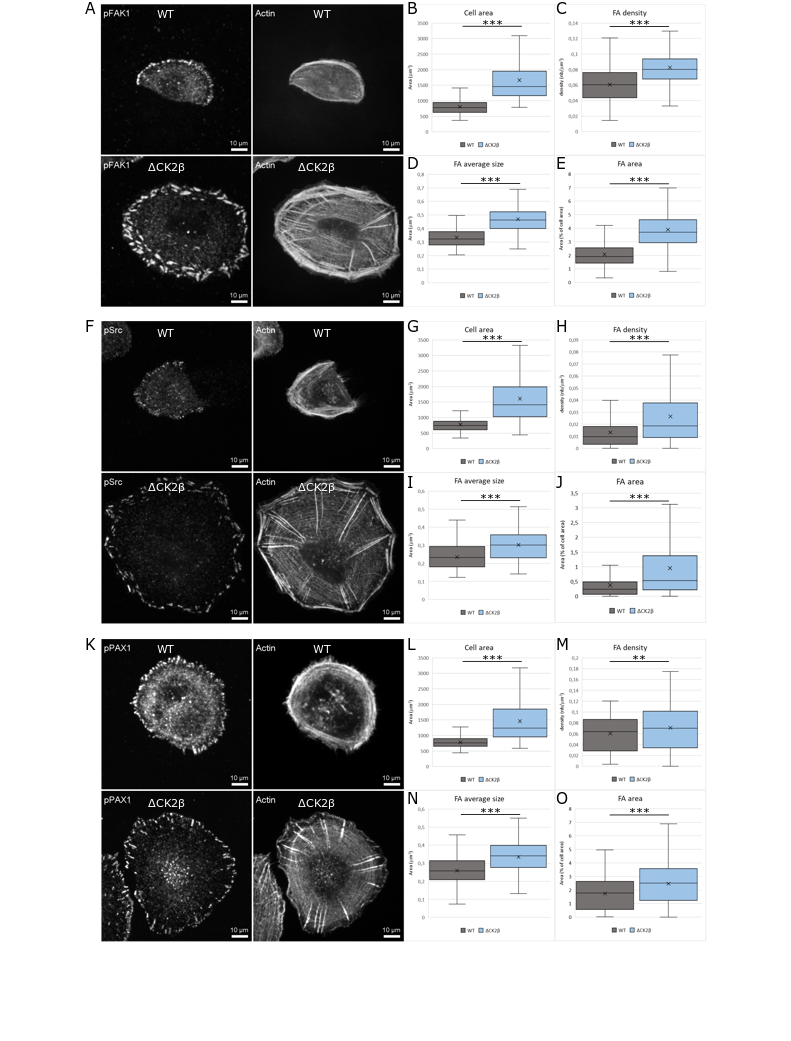

Supplement: Supplementary file 1 [file DataSheet1.zip › Sup data/Sup Fig 5.png]
